# Targeting the recurrent Rac1P29S neoepitope in melanoma with heterologous high-affinity T cell receptors

**DOI:** 10.3389/fimmu.2023.1119498

**Published:** 2023-02-16

**Authors:** Lena Immisch, George Papafotiou, Nerea Gallarín Delgado, Vivian Scheuplein, Annette Paschen, Thomas Blankenstein, Gerald Willimsky

**Affiliations:** ^1^Institute of Immunology, Charité-Universitätsmedizin Berlin, corporate member of Freie Universität Berlin and Humboldt-Universität zu Berlin, Berlin, Germany; ^2^German Cancer Research Center, Heidelberg, Germany; ^3^German Cancer Consortium, partner site Berlin, Berlin, Germany; ^4^Max Delbrück Center for Molecular Medicine in the Helmholtz Association, Berlin, Germany; ^5^Department of Dermatology, University Hospital Essen, University of Duisburg-Essen, Essen, Germany; ^6^German Cancer Consortium, partner site Essen, Essen, Germany; ^7^Berlin Institute of Health at Charité-Universitätsmedizin Berlin, Berlin, Germany

**Keywords:** neoantigen, TCR gene therapy, melanoma, Rho (Rho GTPase), humanized mouse models

## Abstract

Recurrent neoepitopes are cancer-specific antigens common among groups of patients and therefore ideal targets for adoptive T cell therapy. The neoepitope F**S**GEYIPTV carries the Rac1P29S amino acid change caused by a c.85C>T missense mutation, which is the third most common hotspot mutation in melanoma. Here, we isolated and characterized TCRs to target this HLA-A*02:01-binding neoepitope by adoptive T cell therapy. Peptide immunization elicited immune responses in transgenic mice expressing a diverse human TCR repertoire restricted to HLA-A*02:01, which enabled isolation of high-affinity TCRs. TCR-transduced T cells induced cytotoxicity against Rac1P29S expressing melanoma cells and we observed regression of Rac1P29S expressing tumors *in vivo* after adoptive T cell therapy (ATT). Here we found that a TCR raised against a heterologous mutation with higher peptide-MHC affinity (Rac2P29L) more efficiently targeted the common melanoma mutation Rac1P29S. Overall, our study provides evidence for the therapeutic potential of Rac1P29S-specific TCR-transduced T cells and reveal a novel strategy by generating more efficient TCRs by heterologous peptides.

## Introduction

Adoptive T cell therapy (ATT) as a treatment option against cancer is coming of age, so far primarily with remarkable efficiencies against non-solid leukemia and lymphoma, for example using chimeric antigen receptor modified T cells (CAR-Ts) targeting the lineage-specific surface protein CD19 (1). Targeting cancer mutations by reactivating neoepitope-specific T cells using checkpoint blockade has shown therapeutic success in half of the patients harboring solid cancers with high mutational load but is hampered in patients with tumors carrying lower numbers of mutations (2). Therefore, TCR gene therapy, the genetic modification of autologous patient T cells by introducing a therapeutic TCR and thus grafting of new antigen specificities onto patients’ T cells, may be a valuable alternative. Since TCR gene therapy allows for targeting proteins independent of cellular localization it broadens the spectrum of target antigens. Therefore, it also allows for targeting somatic mutations, so called neoantigens, that come with the best possible risk-benefit ratio because these are truly cancer-specific mutant antigens not expressed in normal tissue (3).

Rac (Ras-related C3 botulinum toxin substrate) proteins are a subfamily of the Rho family of GTPases involved in many cellular processes including cell migration, cytoskeleton reorganization and cell transformation (4). Due to its role to control a variety of cellular functions, aberrant Rac signaling is often involved in tumorigenesis (5, 6). The Rac family comprises the homologous proteins Rac1, Rac2 and Rac3; this study focused on Rac1 and Rac2, for which point mutations in tumors have been described. A single-nucleotide variant (SNV) at position 85C>T leads to the Rac1P29S amino acid change with a strong UV signature. Following mutations in Braf V600 and Nras Q61, Rac1 P29 is the third most commonly mutated protooncogene in cutaneous melanomas and with up to 9% of sun-exposed melanomas carrying this mutation the most common cancer-associated recurrent missense mutation among the family of small Rho GTPases (7, 8). The Rac1 mutation occurred in both Braf and Nras mutant melanomas (7, 8), but a higher percentage of Braf/Nras wild type melanomas possess the Rac1P29S mutation (9). In addition, both Braf and Nras mutations also occur in benign naevi and seem insufficient to cause progression towards melanoma (10), altogether suggesting that Rac1P29S could also be a driving event independently of these oncogenes (11). Additionally, the mutation confers resistance to Braf inhibition by vemurafenib and dabrafenib *in vitro*, suggesting a role of Rac1P29S mutation as a biomarker for Raf inhibitor resistance in melanoma patients (12). It has furthermore been reported that Rac1P29S upregulates PD-L1 expression in melanoma (13), and thus may contribute to immune evasion. Due to its importance in proliferation, metastasis and drug resistance, Rac1 is an important therapeutic target in melanoma, but so far is considered undruggable, which makes targeting Rac1P29S in melanoma a challenge.

Other Rho GTPase members also harbor mutations in homologous residues, but these are less frequently found in tumorigenesis (<1% incidence), such as Rac2 (P29L) and Rhot (P30L) (8). The P29L (c.86C>T) mutation in Rac2 was not only detected in melanoma but also in a breast cancer samples, confirming an important role of the P29 position in oncogenesis (14, 15).

Since mutant Rac1 is specifically expressed on cancer cells and is important for the perpetual growth and survival of tumor cells, it is a promising target candidate for TCR gene therapy, a methodology that equips patient T cells with anti-cancer specificity. Additionally, the mutation is found in a large percentage of cancer patients and presented by a frequent HLA-molecule. Here, we explore the Rac1P29S mutation together with the less frequent Rac2P29L mutation as targets for adoptive T cells therapy.

## Materials and methods

### Peptide immunization of mice

Mutation-specific T cells were generated in ABabDII mice expressing a diverse human TCR repertoire restricted to HLA-A*02:01 (16). The mice are additional deficient for mouse TCR and mouse MHC I expression. Mice were immunized by subcutaneous injection of 100 μg Rac1P29S (F**S**GEYIPTV), Rac2P29L (F**L**GEYIPTV) or RhotP30L (F**L**EEVPPRA) peptide in a 1:1 solution of incomplete Freund’s adjuvant and PBS containing 50 μg CpG. After priming, the mice received the same immunization twice as boosts in a three weeks interval. To assess CD8^+^ T cell responses, peripheral T cells were restimulated *in vitro* with either 10^-6^ M peptide, PBS as a negative control, or 10^6^ Dynabeads mouse T activator CD3/CD28 (Gibco) as a positive control. After 2h, Brefeldin A (BD) was added to the cultures and after overnight culturing, specific CD8^+^ T cells were measured by intracellular IFNγ staining (PE anti-mouse IFNγ XMG1.2, Biolegend).

### Isolation and cloning of TCRs

To isolate specific TCRs, immunized mice were sacrificed; splenocytes and lymphocytes from inguinal lymph nodes were prepared and CD4^+^ T cells were depleted using microbeads (Miltenyi Biotec). 1x10^6^ splenocytes were cultured in T cell media (TCM, RPMI (Gibco™) containing 10% FCS (Pan Biotech), 1 mM HEPES (Gibco™), 100 IU/ml PenStrep (Gibco™), 50 μM 2-Mercaptoethanol (Gibco™)) supplemented with 100 IU/ml IL-2 (Peprotech) for 10 days in the presence of 10^-8^ M or 10^-9^ M peptide. Reactive T cells were either sorted using a Rac1-specific tetramer (pA2-tetramer, Beckman Coulter) or the mouse IFNγ secretion assay (Miltenyi). Four hours prior to the *in vitro* assessment of IFNγ secretion, cells were stimulated with a peptide concentration of 10^-6^ M. To sort IFNγ-secreting CD8^+^ T cells, cells were stained with anti-mouse CD3-APC (145-2C11, Biolegend) and anti-mouse CD8-PerCP (53-6.7, Biolegend) at 4°C for 30 minutes. For sorting of tetramer-positive T cells, staining was done with PE-labeled pA2-tetramer, anti-mouse CD3-APC and anti-mouse CD8-PerCP. T cells were subsequently sorted (BD FACS Aria III) into RTL lysis buffer for RNA isolation with RNeasy Micro Kit (QIAGEN). SMARTer™ RacE cDNA Amplification Kit (Clontech Laboratories) was used to synthesize first-strand cDNA synthesis and 5’-RACE PCR. The TCR sequence was specifically amplified using 0.1 μM universal primer (5’-ctaatacgactcactatagggcaagcagtggtatcaacgcagagt-3’) and either 0.1 μM hTRAC (5’-cggccactttcaggaggaggattcggaac-3’) or hTRBC (5’−ccgtagaactggacttgacagcggaagtgg-3’) specific primer and 1U Phusion^®^ HotStart II polymerase (Thermo Scientific) from 1-2 μl of the reverse transcriptase reaction. The amplicons were analyzed on an agarose gel and specific bands were cut out and cloned using a Zero Blunt^®^ TOPO^®^ PCR cloning kit (Life Technologies). A T3 primer (5’-aattaaccctcactaaaggg-3’) was used to sequence plasmids from isolated individual clones (Eurofins Genomics). Dominant TCR-α/β chains were selected and corresponding TCR-α/β chains were linked using a P2A element and constant regions of the TCRs were exchanged with mouse constant regions. The codon-optimized TCR cassettes were synthesized by GeneArt (Thermo Fisher Scientific) and cloned into an MP71 vector.

### Plasmid constructs and cDNA synthesis

All retroviral packaging plasmid vectors were based on plasmid MP71 (17). Plasmids MP71-A2 encoding HLA-A*02:01, and MP71-CDK4-R24L-i-GFP encoding the full-length cDNA of CDK4 harboring the R24L mutation and co-expressing EGFP through an IRES element, were a kind gift from M. Leisegang. To construct MP71-CDK4R24L-P2A-GFP, a P2A-EGFP fragment was PCR amplified from plasmid pcDNA3.1-Hygro(^+^)-M7PG (kind gift V. Anastasopoulou) using primer CDK4-P2A_F (5’-acataaggatgaaggtaatccggagggcagcggcgccacc aac-3’) combined with GFP-PRE_Rev (5’-aatggcggtaagatgctcgaatttcatttgtacagctcgtccatgc-3’). Produced amplicons bear homologies to the above plasmids and can serve to prime overlap extension PCR (OE-PCR), replacing the IRES-EGFP elements with P2A-EGFP. In brief, the respective OE-PCR amplicon was mixed in a 200 molar excess ratio to 10 ng of MP71-CDK4-R24L-i-GFP, in 25 μl PCR reactions and were cycled, using 52°C annealing temperature and a 7-minute extension time at 72°C, for 21 cycles. Reactions were subsequently digested with DpnI and transformed into competent E. coli. All PCRs described were performed using Q5, High fidelity 2x Master mix (NEB). Unless stated otherwise, all other constructs were synthesized by GeneArt Gene Synthesis, ThermoFischer Scientific and are followed by an AAY sequence and EGFP.

### Cells and cell culture

The retroviral packaging cell lines 293GP-GLV (producing amphotropic retroviral vectors) and Plat-E (producing ecotropic retroviral vectors) were cultured in DMEM supplemented with 10% FCS (18, 19). TAP-deficient T2 cells (RRID : CVCL_2211, ATCC: CRL-1992) and human PBMCs were cultured in T cell media (TCM, RPMI (Gibco™) containing 10% FCS (Pan Biotech), 1 mM HEPES (Gibco™), 100 IU/ml PenStrep (Gibco™), 50 μM 2-Mercaptoethanol (Gibco™). Mouse T cells were cultured in mouse T cell media (mTCM, RPMI (Gibco™) containing 10% FCS (Pan Biotech), 100 IU/ml PenStrep (Gibco™), 50 μM 2-Mercaptoethanol (Gibco™) and Sodium Pyruvate). The cell lines Mel624 (RRID : CVCL_8054), UKRV-Mel-21a (referred to hereafter as Mel21a) (20), Mel20aI (RRID : CVCL_A157), MaMel085 (called here Mel085) (RRID : CVCL_A220), Mel55b (RRID : CVCL_A190), HepG2 (RRID : CVCL_0027), SH-SY5Y (RRID : CVCL_0019), were cultured in RPMI (Gibco™) supplemented with 10% FCS (Pan Biotech) and 100 IU/ml PenStrep (Gibco™). MC703 cells were kindly provided by M. Leisegang and are described in (21). The panel of EBV–transformed lymphoblastoid B cell lines [LCLs (22)] were cultured in RPMI supplemented with 10% FCS, 1x antibiotic-antimycotic, 1mM sodium pyruvate and 1x non-essential amino acids.

### Retroviral transduction of TCRs into primary T cells

TCR gene transfer was carried out as described before (17, 23). In brief, for retrovirus generation, 293GP-GLV cells were transfected with MP71 vector carrying the respective TCR cassettes using Lipofectamine 3000 (ThermoFisher Scientific). On the same day, PBMCs from healthy donors were seeded on plates coated with 5 μg/ml anti-CD3 (OKT3, Invitrogen) and 1 μg/ml anti-CD28 antibodies (CD28.2, Invitrogen) in TCM supplemented with 100 IU/ml IL-2 (Peprotech). 48 hours later, the virus supernatant was harvested, filtered and supplemented with 8 μg/ml protamine sulfate (Sigma-Aldrich) and 100 IU/ml IL-2, before spinoculation with the activated T cells at 800g for 90 minutes at 32°C was performed. The next day, a second supernatant was harvested from the same 293GP-GLV cells, transferred to a RetroNectin (Takara Bio) coated plate and centrifuged at 3200g for 90 minutes at 4°C. The PBMCs were harvested, supplemented with 100 IU/ml IL-2 and 8 μg/ml protamine sulfate and spinoculated with the virus-containing plates at 800g for 30 minutes at 32°C. After the second transduction, T cells were expanded for 10 days, before being transferred to low IL-2 (10 IU/ml). After 48 hours, transduced T cells were harvested, analyzed for TCR expression by flow cytometry and frozen for future experiments. To detect the transduction rate of the TCRs transduced into primary T cells, the following antibodies were used in a 1:100 dilution at 4°C for 30 min: anti-human CD3-PerCP (UCHT1, Biolegend), anti-human CD8-APC (HIT8a, Biolegend) and anti-mouse TCR β chain-PE (H57-597, Biolegend).

For mouse transductions, Plat-E cells were transfected with MP71 vector carrying the respective TCR cassettes using Lipofectamine 3000 (ThermoFisher Scientific). On the following day, spleen cells were isolated from HHD mice (24) and erythrocytes were lysed by ammonium chloride treatment. 2x10^6^/ml cells were incubated in mTCM supplemented with 1 μg/ml anti-mouse CD3, 0.1 μg/ml antimouse CD28 antibodies (BD Biosciences (BD), Franklin Lakes, NJ, USA) and 10 IU/ml human IL-2 (Proleukin S, Novartis, Basel, Switzerland). On the next day, 1x10^6^ cells were transduced by spinoculation in 24-well non-tissue culture-treated plates pre-coated with 12.5 μg/ml RetroNectin (TaKaRa, Otsu, Japan) and virus particles (3200 x g, 90 min, 4°C) in 1ml mTCM supplemented with 10 IU/ml IL-2 and 4x10^5^ mouse T-Activator beads (Life Technologies). A second transduction was performed on the following day by spinoculation with 1 ml virus supernatant (+ 10 IU/ml IL-2). T cells were expanded in mTCM (+ 50 ng/ml IL-15 (Miltenyi Biotec) for 10 days. TCR transduction rate was measured by flow cytometry using the following antibodies in a 1:100 dilution at 4°C for 30 min: anti-mouse CD3-BV421 (UCHT1, Biolegend), anti-mouse CD8-APC (HIT8a, Biolegend) and FITC-labeled anti-human TCR Vβ22 (IMMU 546, Beckman Coulter), Vβ9 (MKB1, Biolegend) and Vβ1 (BL37.2, Beckman Coulter) for TCRs 22894, 5934 and 14/35, respectively. These antibody combinations were also used to stain blood from adoptively transferred HHDxRag^-/-^ mice [ (21); see below].

### Retroviral transduction of tumor cell lines

Mel085-A2 and SH-SY5Y-A2 cells were generated by transfecting MP71-HLA-A*02:01 into 293GP-GLV cells using Lipofectamine 3000 (ThermoFisher Scientific). 48 hours after transfection, 293GP-GLV virus-containing supernatant was harvested, supplemented with 8 μg/ml protamine sulfate and transferred to the respective glioma cell line. Cells and virus supernatant were spinoculated at 800g for 90 minutes at 32°C. The medium was changed to the respective growth medium 6 hours later. To analyze successful transduction, flow cytometry was used to determine the fraction of GFP-positive cells. HLA-A*02:01 transduction was confirmed by flow cytometry using an anti-human HLA-A*02:01-APC (BB7.2, Biolegend) specific antibody and an APC mIgG2b, k isotype control (MPC-11, Biolegend). Mel55, Mel085-A2 and Mel20aI cells expressing CDK4R24L-GFP were generated by retroviral transduction using MP71 plasmid expressing the R24L mutant CDK4 full-length cDNA followed by a P2A element and GFP. MC703 cells were transduced with a triple epitope construct of Rac1P29S nonamer separated by AAY proteasomal cleavage sites tagged with GFP (5’LTR – (P29S)_3_ – GFP – PRE – 3’LTR).

### Co-culture experiments

All co-culture experiments to detect IFNγ secretion were conducted using 1x10^4^ - 1x10^5^ transduced T cells with 1x10^4^ -1x10^5^ target cells as indicated for 22-24 hours in a 96 well plate. As a positive control for the T cell activation, 50 ng/ml PMA (Phorbol-12-myristat-13-acetat, (Calbiochem) and 1 μg/ml Ionomycin (Calbiochem) were added to the transduced T cells. T2 cells were loaded with the labelled peptides (JPT Peptide Technologies GmbH) at the indicated concentrations. Secreted IFNγ amounts in the supernatant were measured by ELISA (BD OptEIA; BD Biosciences). Alanine-exchanged Rac1/2 peptides (all JPT Peptide Technologies GmbH, >95% purity) were added at 10^-5^ M or 10^-9^ M.

### Cytotoxicity assay

The cytotoxic potential of transduced T cells was analyzed using the live cell imaging system IncuCyte Zoom (Essen Bioscience). 3-5x10^3^ GFP positive target cells were resuspended in TCM without phenol red and seeded into flat-bottom 96 well plates. The following day, transduced T cells in a 5:1 or 15:1 (effector: target) ratio were added to the respective wells in triplicates. GFP expression in target cells was determined every hour over a time period of 72 hours at 37°C and 5% CO2. For analysis, the average of GFP total area (μm²/image) in the target cells co-cultured with the respective TCR-transduced T cells was calculated and normalized to the average of GFP total area (μm²/image) of the same target cells co-cultured with mock-transduced T cells (% of mock T cells).

### Tumor challenge and adoptive T cell transfer

12-20 weeks old HHDxRag^-/-^ mice (21) were injected with 1x10^6^ MC703-FSG tumor cells and tumor growth was measured 2-3 times a week. When tumors reached a tumor size of 300-1000 mm^3^ (mean treatment group tumor size ~500 mm^3^), mice were intravenously injected with TCR-engineered T cells obtained from HHD mice in 100µl PBS (adjusted to 1x10^6^ CD8^+^TCR^+^ HHD T cells per mouse). Tumor volume was determined by caliper measurement of the tumor parameters (x,y,z) according to the formula (xyz)/2. Mice were sacrificed and tumors isolated when tumors reached the maximum tolerable size.

### Statistical analysis

Statistical analysis was conducted using GraphPad prism software. Statistics (standard deviation (SD), t-test, two-way ANOVA) are indicated in the figure legends. If not stated otherwise, significance is given as ns = not significant, p<0.001 *** and p<0.0001 ****.

## Results

### High affinity Rac1/2-specific TCRs were successfully isolated after peptide immunization

The Rho GTPase mutations, Rac1P29S and Rac2P29L and RhotP30L create epitopes (Rac1P29S_28-36_ F**S**GEYIPTV, Rac2P29L_28-36_ F**L**GEYIPTV and RhotP30L_29-37_ F**L**EEVPPRA) predicted to bind HLA-A*02:01 with high affinity of 18.2 nM, 2.3 nM and 27.7 nM, respectively (**Table 1**) (25). Peptides binding to MHC class I molecules are defined as strong binders with an IC_50_ value <50 nM (26, 27). In contrast to the wildtype peptide, the Rho GTPase mutant epitopes are predicted strong binders, with the less frequent mutant Rac2P29L epitope being the strongest.

**Table 1:** Prediction of binding of peptides to MHC class I molecules [NetMHC 4.0 DTU Health Tech (25)].

	Peptide	HLA	Affinity (nM)
**Rac1P29S**	F**S**GEYIPTV	HLA-A*02:01	18.2
**Rac2P29L**	F**L**GEYIPTV	HLA-A*02:01	2.3
**Rac1/2 wt**	F**P**GEYIPTV	HLA-A*02:01	573.0
**RhotP30L**	F**L**EEVPPRA	HLA-A*02:01	27.7
**Rhot wt**	F**P**EEVPPRA	HLA-A*02:01	21063.3

**Figure 1:**
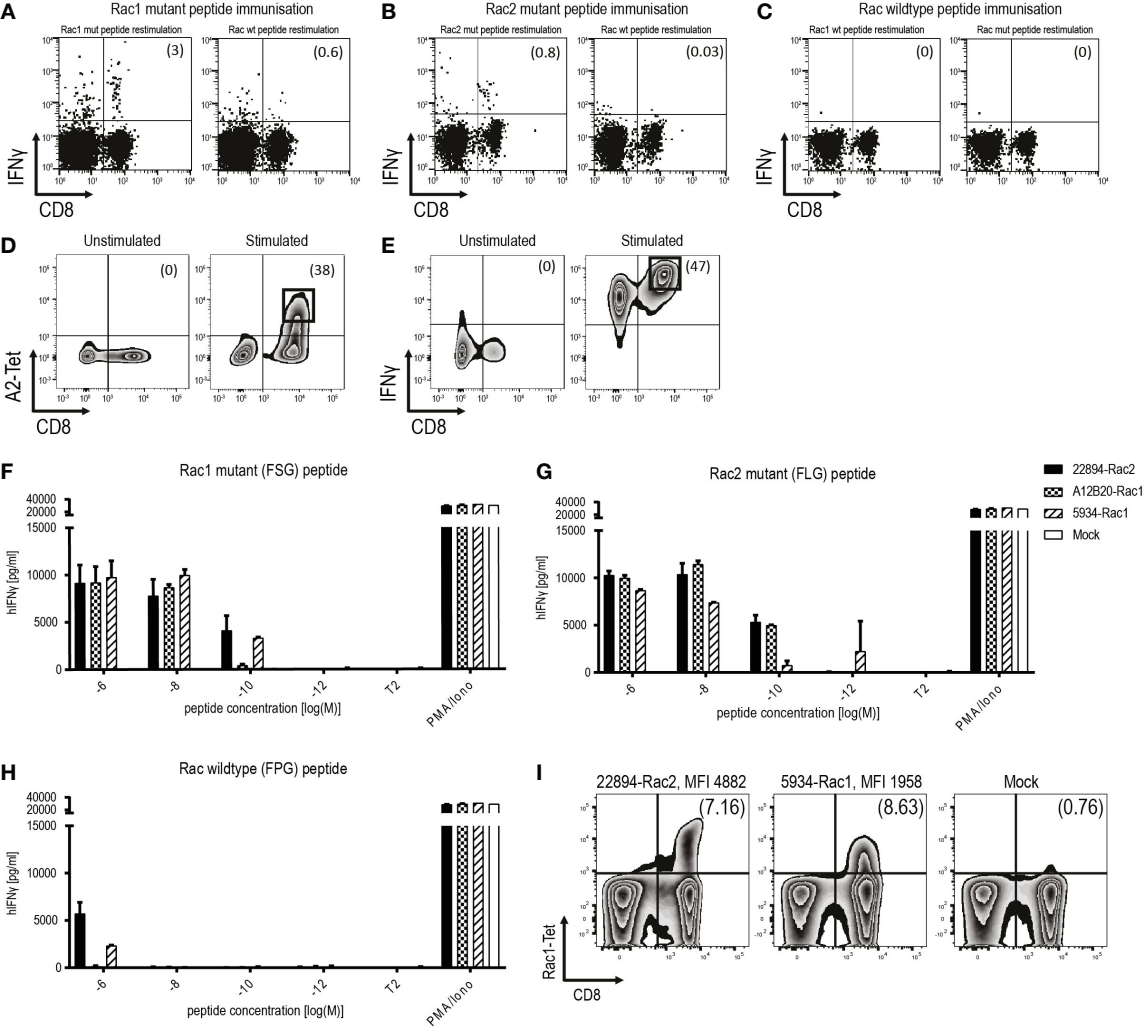
Identification and isolation of mutant Rac1/2-specific, high-affinity TCRs in ABabDII mice. **(A-C)** Representative examples of *ex vivo* intracellular IFNγ staining of peripheral T cells obtained from ABabDII mice immunized with **(A)** Rac1P29S mutant peptide, **(B)** Rac2P29L mutant peptide or **(C)** Rac wild type peptide. Splenocytes were restimulated with the indicated peptide 7 days after the last immunization of ABabDII mice. Numbers in brackets represent percent of CD8^+^ T cells **(D)** Representative pA2 tetramer staining of mutant Rac1-specific CD8^+^ T cells 10 days after spleen cell culture in the presence of 10^-9^ M mutant Rac1 peptide. Cells were gated on lymphocytes and CD3^+^ T cells. Numbers in brackets represent percent of pA2 tetramer^+^ CD8^+^ T cells, unstimulated splenocytes served as a negative control. Sorted cells are depicted in squares. **(E)** Identification of IFNγ^+^ CD8^+^ T cells using IFNγ-capture assay depicted by representative staining of mutant Rac1-specific CD8^+^ T cells 10 days after spleen cell culture in the presence of 10^-8^ M mutant Rac1 peptide. Cells were gated on lymphocytes and CD3^+^ T cells, unstimulated splenocytes served as a negative control. Numbers in brackets represent percent IFNγ^+^CD8^+^ T cells. Sorted cells were depicted in squares. **(F-H)** TCR-transduced T cells were co-cultured with peptide-loaded T2 cells (1x10^4^ cells, 1:1 ratio) for 22 hours in triplicates. IFNγ levels were determined in an ELISA assay. PMA and Ionomycin (P/I) stimulation served as a positive control, non-loaded T2 cells as a negative control. T2 cells were loaded with indicated concentrations of **(F)** Rac1 mutant peptide (F**S**GEYIPTV), **(G)** Rac2 mutant peptide (F**L**GEYIPTV) or **(H)** Rac wild type peptide (F**P**GEYIPTV). **(I)** Affinity of the TCRs to its peptide-MHC complex was determined using a mutant Rac1-specific pA2 tetramer. Binding to the pA2 tetramer is indicated by mean fluorescence intensity (MFI). The experiment was performed three times with similar results and graphs represent means of triplicate cultures ± SD.

To study the potential of the recurrent Rac1P29S mutation as a target for ATT, we isolated and characterized TCRs against Rac1P29S. Additionally, TCRs were also raised against Rac2P29L, a mutation that differs in one amino acid but has a higher predicted peptide-MHC affinity than Rac1P29S. Mutant peptides were used to immunize ABabDII mice, a transgenic mouse model expressing an HLA-A*02:01-restricted diverse human TCR repertoire (16). Peripheral T cells of Rac1 and Rac2 mutant peptide immunized mice showed an immune response upon restimulation, measured by intracellular IFNγ responsiveness (**Figures 1A–C**). Repeated immunization of ABabDII mice with the RhotP30L peptide-epitope did not result in a CD8^+^ T cell response (data not shown). To isolate specific TCRs, we cultured splenocytes of responsive mice in the presence of the respective mutant peptide for 10 days and sorted either peptide HLA-A*02:01 tetramer positive (pA2 tetramer^+^) T cells (**Figure 1D**) or IFNγ^+^ CD8^+^ T cells (**Figure 1E**). We identified dominant α and β chains of the responsive mice #22894 (Rac2 mutant immunized), #A12B20 and #5934 (Rac1 mutant immunized) (**Table 2**). After replacing of the human constant regions by murine ones in order to reduce mispairing between endogenous and transduced TCR chains, the constructs comprising TCRβ-P2A-TCRα cloned into pMP71 vector were retrovirally transduced into human T cells of healthy donors. Successful transduction of the three Rac1/2-specific TCRs, as well as a well-characterized CDK4R24L-specific TCR (14/35) as control, was measured by staining for the murine constant β chain in CD8^+^ T cells and analyzed by flow cytometry (**Supplementary Figure 1A**). We found CD8^+^ and TCR^+^ double positive T cells at similar percentages ranging from 26.3% to 34.6% of CD3^+^ T cells.

**Table 2:** List of isolated Rac 1/2 -specific TCRs.

TCR	α chain	Frequency α chain	β chain	Frequency β chain	Immunization
**A12B20**	TRAV12‐2*02 –CAAQSARQLTF –TRAJ22*01	3/12	TRBV20‐1*01(/02) –CSARDLITDTQYF –TRBJ2‐3*01	7/11	Rac1P29S_28-36_
**5934**	TRAV13-1*01–CAASRGGAQKLVF –TRAJ54*01	12/15	TRBV3-1*01 –CASSQLAGGPLYNEQFF –TRBJ2-1*01	14/14	Rac1P29S_28-36_
**22894**	TRAV13-1*03 – CAVGANNLFF – TRAJ39*01	8/13	TRBV2*01 – CAASMGNAGNMLTF – TRBJ2-7*01	10/12	Rac2P29L_28-36_

Name of TCR, frequency and details of the alpha and beta chains as well as the CDR3 region are listed.

To determine the functional avidity of the isolated TCRs, TAP-deficient T2 cells were loaded with titrated amounts of Rac1 mutant (FSG) peptide (**Figure 1F**), Rac2 mutant (FLG) peptide (**Figure 1G**) and as a negative control Rac wild type (FPG) peptide (**Figure 1H**). The two TCRs isolated after Rac1 mutant peptide immunization showed high affinity to their respective peptide down to a concentration of 10^-9^ M for TCR A12B20-transduced, and 10^-10^ M for TCR 5934-transduced T cells. Interestingly, also the TCR 22894, isolated after immunization with the Rac2 mutant peptide showed high functional avidity towards the Rac1 mutant peptide down to a concentration of 10^-10^ M. When loaded with the Rac2P29L peptide, the mutant Rac1-specific TCRs also recognized the Rac2 mutant peptide (**Figure 1G**). The Rac wild type (FPG) peptide was only recognized when co-cultured with the T cells in the highest peptide concentrations of 10^-6^ M. Since the Rac2P29L mutation is less common in human cancers, the subsequent experiments focused primarily on the Rac1P29S mutation as a target for ATT. To determine the binding strength of the TCRs to the Rac1 peptide-MHC (pMHC) complex, we stained the two TCRs 22894 and 5934 that performed best in the affinity assays with a mutant Rac1-specific pA2 tetramer. As depicted in **Figure 1I**, the heterologous Rac2-specific TCR 22894-transduced T cells showed a higher MFI of 4889 compared to the Rac1-specific TCR 5934-transduced T cells (MFI 1958).

### Mutant Rac1/2-specific T cells showed cytotoxicity against melanoma cell lines naturally expressing mutant Rac1

**Figure 2:**
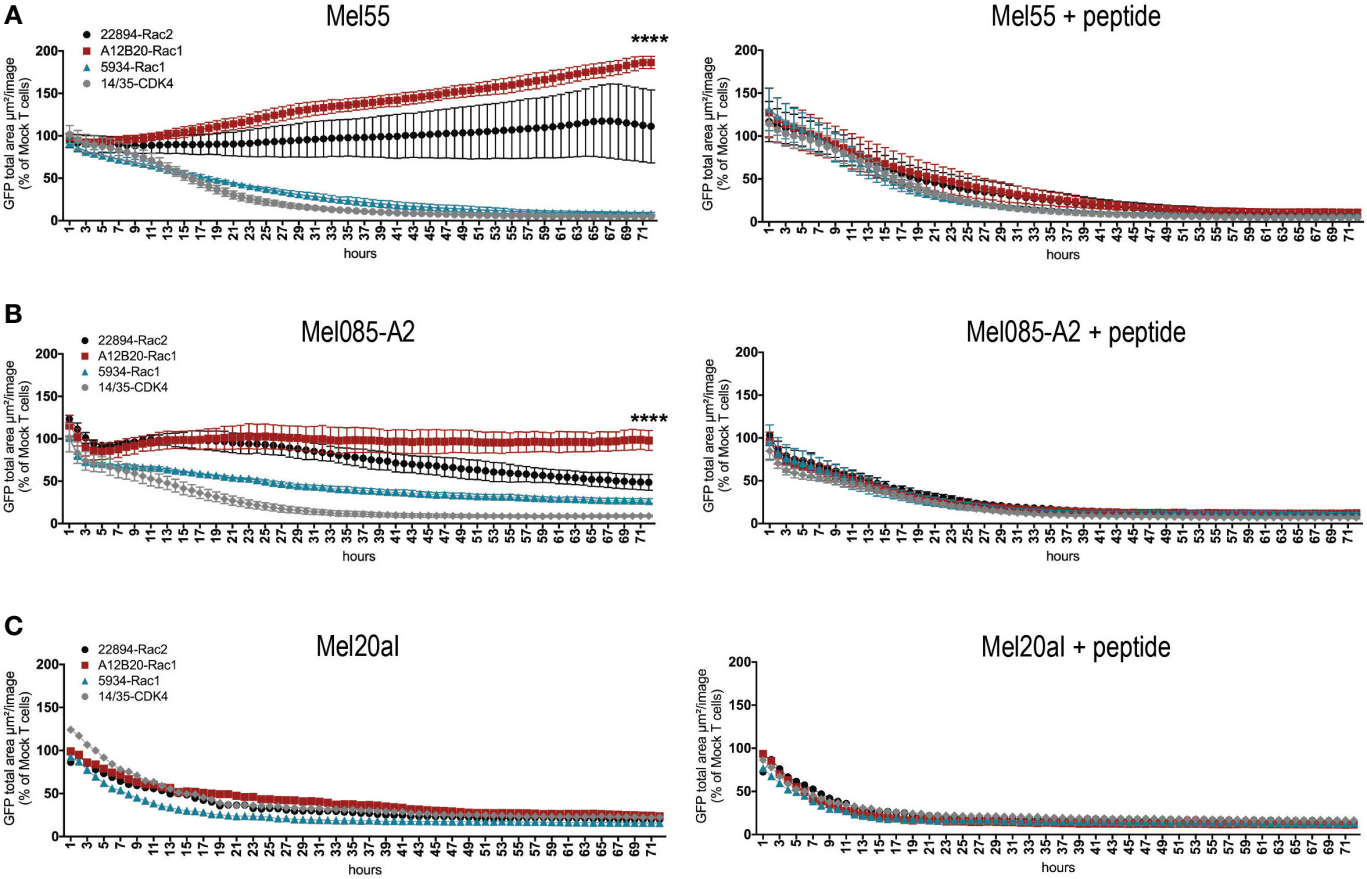
Rac1/2-specific T cells showed cytotoxicity against melanoma cell lines naturally expressing Rac1P29S. **(A-C)** 15x10^3^ transduced CD8^+^ T cells were co-cultured as triplicates at a 5:1 E:T ratio with **(A)** Mel55 cells, **(B)** Mel085-A2 and as singlets at a 15:1 E:T ratio with **(C)** Mel20aI cells. Right panels show the same cell line loaded with 10^-6^ Rac1 mutant peptide. Cells were retrovirally transduced to express CDK4R24L full-length cDNA. Cytotoxicity was observed over 72 hours using the live cell imaging system IncuCyte Zoom (Essen Bioscience). Values were calculated by normalizing the average GFP total area (µm²/image) in the target cells co-cultured with the respective TCR-transduced T cells to the average of that co-cultured with mock transduced T cells. The experiment was performed three times with similar results, for triplicate cultures graphs represent means ± SD. Cytotoxicity was compared at 72h using two-way ANOVA, only significant results are depicted.

Next, we aimed to confirm the recognition of tumor cell lines that endogenously express the respective mutation. Presence of the Rac1P29S mutation in cell lines Mel55, Mel085, and Mel20aI has been confirmed by Sanger sequencing, interestingly Mel55 has lost the Rac wild type allele (data not shown). All tumor cells were GFP positive, as they were retrovirally transduced to express the positive control CDK4R24L coupled to GFP (**Supplementary Figure 2A**). Since Mel085 melanoma cells are HLA-A*02:01 negative, they were in addition retrovirally transduced to express HLA-A*02:01 as confirmed by flow cytometry (**Supplementary Figure 2B**). The other two cell lines are naturally HLA-A*02:01 positive. To analyze T cell reactivity against the natural occurring Rac1P29S mutation, the mutant Rac1 harboring melanoma cells were co-cultured with TCR-transduced T cells over 72 hours and cytotoxicity was measured by the decrease in GFP expressing target cells determined by live-cell imaging. Rac1-specific TCR 5934-transduced T cells showed cytotoxicity against all three cell lines (**Figures 2A–C**, left panels). The Mel085-A2 cells were also partially lysed by the Rac2-specific 22894 TCR-transduced T cells (**Figure 2B**, left panel), whereas all three Rac1/2-specific TCR-transduced T cell groups (TCRs 5934, A12B20 and 22894) were able to elicit cytotoxicity against Mel20aI cells (**Figure 2C**, left panel). The positive control CDK4-specific TCR 14/35-transduced T cells (28) lysed all CDK4R24L overexpressing target cells (**Figures 2A–C**, left panels). These experiments prove that the melanoma cell lines generally are able to process and present neoantigens. It further shows that the *in silico* predicted Rac1P29S epitope similarly is naturally processed and recognized by TCR-redirected T cells, which also could be confirmed when recombinantly mutant Rac1 cDNA was expressed in tumor cells (data not shown). As a positive control, all three cell lines were exogenously loaded with mutant Rac1P29S peptide. It showed that these peptide-loaded cells were efficiently recognized and lysed by the Rac1/2-specific TCR-transduced T cells (**Figures 2A–C**, right panels).

### Mutant Rac1P29S triple epitope was recognized by Rac1/2-specific T cells *in vivo*

**Figure 3:**
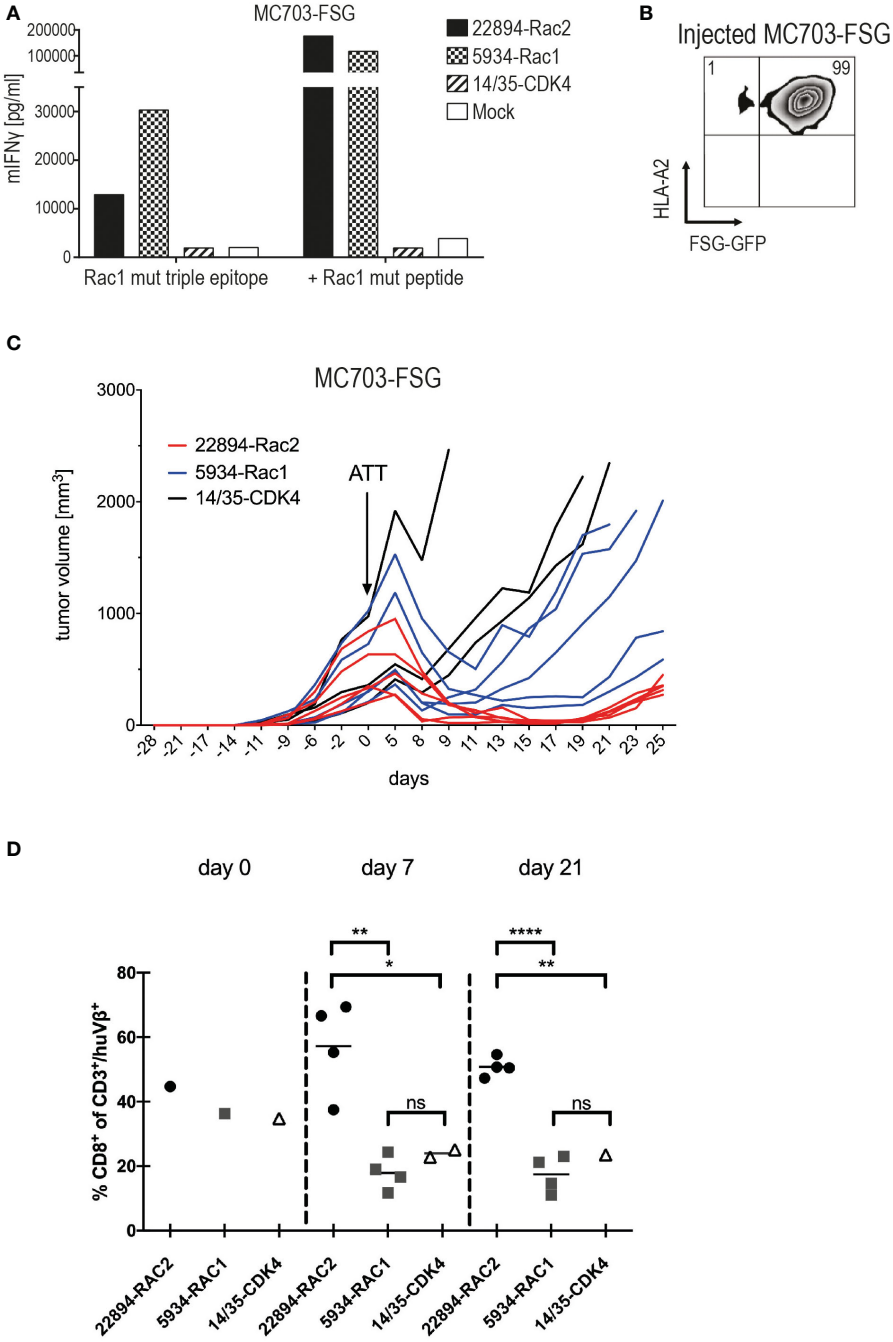
Heterologous Rac2-specific 22894 TCR-transduced T cells elicited tumor regression upon ATT. **(A)** TCR-transduced murine T cells were co-cultured with the mouse tumor cell line MC703 expressing FSG triple epitope (5’LTR - (P29S)_3_ - GFP - PRE - 3’LTR) (1x10^5^, 1:1 ratio) for 24 hours. IFNγ levels were determined in an ELISA assay. Cells were loaded with 10^-6^M Rac1 mutant FSG peptide as a control. The experiment was performed at least three times, one representative experiment is shown. **(B)** FSG-GFP and HLA-A*02:01 expression of MC703-FSG cells before injection measured by flow cytometry. Number indicates percentage. **(C)** 1x10^6^ MC703-FSG cells were injected into HHDxRag^-/-^ mice. Rac1/2-specific T cells were injected 28 days after tumor inoculation (arrow) at an average tumor size of 472 mm^3^ (n=5, 22894-Rac2, red lines), 514 mm^3^ (n=5, 5934-Rac1, blue lines) and 512 mm^3^ (n=3, 14/35-CDK4, black lines), respectively. The experiment was performed two times with similar results (**Supplementary Figure 3A**), standard deviation (SD) and significance is shown in **Supplementary Figure 3C**. **(D)** Mutant Rac2- but not Rac1-specific TCR gene-modified T cells show rapid amplification upon recognition of Rac1P29S^+^ FSG-GFP tumor cells. 22894, 5934 and 14/35 TCR-transduced T cells were identified by staining with anti-human Vβ22, Vβ9 and Vβ1 antibodies, respectively, and number of CD8^+^/huTCR^+^ T cells within the adoptively transferred CD3^+^/huTCR^+^ T cells was calculated. Of note, CD4^+^/huTCR^+^ T cells do not recognize Rac1P29S target cells (data not shown). Treatment groups were compared by unpaired t-test: d7, ** p=0.0022; * p=0.0378; d21, **** p<0.0001; ** p=0.0038; ns = not significant.

The two Rac1/2-specific TCRs that performed best *in vitro*, Rac2P29L-specific TCR 22484 and Rac1P29S-specific TCR 5934, were used to evaluate their ability to reject tumors *in vivo*. The fibrosarcoma cells MC703 (21), which were generated in an HLA-A*02:01-transgenic mouse (HHD, chimeric HLA-A*02:01/H-2D^b^) (24), were transduced with the Rac1P29S triple epitope F**S**GEYIPTV coupled to GFP. Recognition of these cells was confirmed *in vitro* with and without loaded Rac1 mutant peptide (**Figure 3A**). As shown before, both TCR-transduced T cells recognized the target, recognition by Rac1-specific TCR 5934 was slightly higher compared to the heterologous Rac2-specific TCR 22894. Before the MC703-FSG cells were injected into HHDxRag^-/-^ mice, the expression of FSG-GFP and HLA-A*02:01 was determined by flow cytometry (**Figure 3B**). Notably, 99% of injected cells were double positive. When tumors reached an average size of 300-500 mm^3^, mice were treated with 1x10^6^ TCR-transduced HHD T cells. As a negative control, mice were also treated with the irrelevant CDK4-specific TCR 14/35 or left untreated. As depicted in **Figure 3C** and **Supplementary Figure 3A** (respective SD and significance depicted in **Supplementary Figures 3C, D**), Rac1/2-specific 22894 and 5934 TCR-transduced T cells were able to induce regression, while CDK4-specific 14/35 TCR-transduced T cells treated tumors progressively grew after ATT. Interestingly, the heterologous Rac2-specific 22894 TCR-transduced T cells showed greater efficacy in tumor regression compared to the Rac1-specific TCR 5934. To investigate the differences in therapeutic outcome when targeting Rac1P29S^+^ MC703-FSG tumors with either Rac1- or Rac2-specific T cells, we monitored the human TCR-transduced HHD^+^ T cells after transfer into tumor-bearing mice in the second experiment (**Figure 3D** and **Supplementary Figures 3A, B**). On day 7 after ATT high numbers of CD8^+^ Vβ22^+^ 22894 T cells were detected, while T cell expansion of CD8^+^ Vβ9^+^ 5934 T cells was significantly lower. Similarly, target antigen irrelevant CD8^+^ Vβ1^+^ 14/35 T cells showed no amplification on day 7. When analyzing transferred T cells on day 21 it showed that 22894 T cells persisted at a significant higher level in comparison to 5934 and irrelevant 14/35 T cells. Of note, only CD8^+^ but not CD8^–^ human TCR^+^ T cells expanded *in vivo* in response to Rac1P29S expressing MC703-FSG tumors (**Supplementary Figure 3B**).

**Figure 4:**
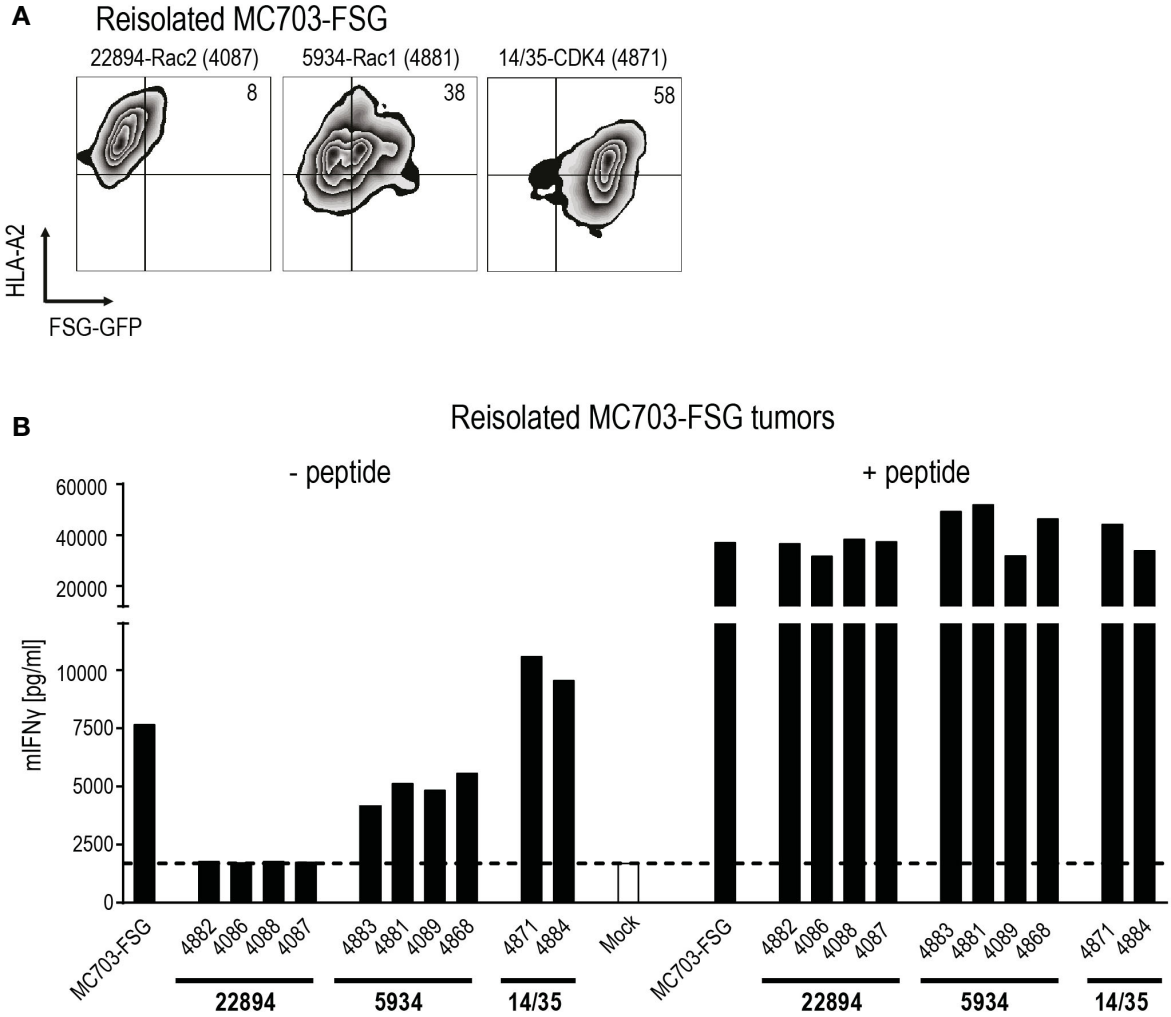
Tumor relapse upon transfer of heterologous mutant Rac2-specific T cells exclusively is due to selection of antigen-negative cancer cells. **(A)** Reisolated tumors were analyzed for HLA-A*02:01 and Rac1P29S FSG-GFP expression by flow cytometry. Numbers in plots indicate percentages. One representative plot per treatment group is depicted, mouse is indicated by numbers in parenthesis. **(B)** Recognition of reisolated tumors was measured in an IFNγ ELISA. Tumors were co-cultured with 5934 TCR-transduced T cells (1x10^5^, 1:1 ratio) for 24 hours. PMA and Ionomycin (P/I) stimulation served as positive control. Cells were loaded with 10^-6^ M Rac1 mutant FSG peptide as control. Mock serves as negative control with no T cells added.

To investigate potential reasons for tumor relapse after initial regression, we reisolated the MC703-FSG tumors and analyzed GFP as well as HLA-A*02:01 expression by flow cytometry (**Figure 4A**). Mice treated with the more efficient Rac2-specific 22894 TCR-transduced T cells showed almost complete loss of FSG-GFP expression down to 8%, while tumors treated with Rac1-specific 5934 TCR-transduced T cells showed partial loss (38% double positive T cells). Tumors treated with CDK4-specific 14/35 TCR-transduced T cells, which did not have any selective pressure on outgrowing FSG-GFP negative tumors, only showed a reduction in HLA-A*02:01 expression. These data suggest that tumors in the 22894 and 5934 TCR treated groups regressed due to target-specific lysis by T cells but subsequently FSG-GFP negative cells led to relapse. As shown in **Figure 3B**, the injected cells were composed of 1% antigen-negative cells, explaining this outgrowth and the selective pressure induced by target-specific TCR-transduced T cells. To confirm this hypothesis, we co-cultured reisolated tumors with a new batch of TCR-transduced T cells (**Figure 4B**). In line with previous data, tumors isolated from mice that were treated with Rac2-specific 22894 TCR-transduced T cells were not recognized by Rac1-specific 5934 TCR-transduced T cells, most likely due to outgrowth of antigen-negative variants. Tumors isolated from Rac1-specific 5934 TCR treated mice were partly recognized, while CDK4-specific 14/35 TCR treated tumors induced comparable IFNγ levels to MC703-FSG control cells which were not previously injected into mice. These *in vivo* data suggest that there might be a potential for ATT with heterologous TCRs that were isolated after immunization with peptides with stronger predicted peptide-MHC binding.

### Recognition pattern and alloreactivity of Rac1/2-specific T cells

To exclude off-target toxicity of the Rac1/2-specific TCRs, we determined their recognition pattern with an alanine scan. To do so, TAP-deficient T2 cells were loaded with Rac1/2 peptides containing single alanine exchanges at concentrations of 10^-9^ M and 10^-5^ M and co-cultured with the respective TCRs. Using a threshold of 50% reduction compared to Rac1/2 unmodified peptide, we identified x(4)-Y-I-P-T-V as the recognition pattern for the TCRs 5934-Rac1 and 22894-Rac2 and F-x(2)-E-x(2)-P-x-V for TCR A12B20-Rac1 (**Supplementary Figures 4A–C**). We found one peptide in the G Protein Subunit Alpha Z (GNAZ) protein (A-A-A-D-Y-I-P-T-V, IC_50 =_ 23.16 nM) with the same recognition pattern as the TCRs 22894-Rac2 and 5934-Rac1. We observed cross-recognition of GNAZ (AAA) peptide in a peptide titration assay (**Supplementary Figure 4D**) and in an assay using a mouse cell line transduced to express a GNAZ triple 35mer (**Supplementary Figure 4E**). However, human cell lines endogenously expressing GNAZ in high amounts (https://www.proteinatlas.org/ENSG00000128266-GNAZ/cell+line) were not recognized by TCR-transduced human T cells (**Supplementary Figure 4F**), demonstrating that this epitope is not processed and presented naturally in sufficient amounts. Since GNAZ is also highly expressed in brain tissue further evaluation of potential cross-reactivity will be necessary (29) to justify clinical application of TCRs 22894-Rac2 and 5934-Rac1 in high-risk patients in order to allow for effective tumor-killing without causing dose-limiting pathology in normal somatic tissues (30). For the Rac1-specific TCR A12B20 we identified a peptide in the Nibrin protein (F-R-I-E-Y-E-P-L-V, IC_50 =_ 439 nM) with the same recognition pattern. Using a peptide titration assay we excluded cross-reactivity of TCR A12B20-Rac1 to this peptide (**Supplementary Figure 4G**).

Since the TCRs were isolated from transgenic mice expressing HLA-A*02:01 but no other HLAs, we tested for MHC alloreactivity using a panel of EBV-transformed lymphoblastoid B cell lines (LCLs) expressing different MHC class I molecules (**Supplementary Table 1**). No allorecognition of the Rac2-specific TCR 22894 (**Supplementary Figure 5A**) and Rac1-specific TCR 5934 (**Supplementary Figure 5B**) was observed using two different donors. In contrast, TCR A12B20-Rac1 transduced T cells recognized the LCL cell lines Bello and WT49. No shared HLA-A, -B or-C between these cell lines was detected, therefore, more research may be required to determine the scope of the allorecognition mediated by TCR A12B20-Rac1 (**Supplementary Figure 5C**).

## Discussion

In this study, we investigated the potential of three Rac1P29S-specific TCRs derived after immunization of human TCR gene loci transgenic mice with peptide-epitopes containing either Rac1P29S or Rac2P29L mutation. We detected high affinity of all three TCRs transduced into human PBMCs against both the mutant Rac1 and the mutant Rac2 peptide-epitope loaded on TAP-deficient T2 cells. This indicates that the one amino acid change in the epitopes is only responsible for the binding affinity to MHC class I complexes (anchor residue) but not to the TCR, which was also confirmed by detecting the TCR recognition pattern by an alanine scan. Therefore, also the heterologous Rac2P29L-derived TCR can potentially be used clinically to target the more frequent melanoma mutation Rac1P29S. By demonstrating cytotoxicity of our TCR-transduced T cells against three melanoma cell lines harboring the Rac1P29S mutation we proved natural processing and presentation of the predicted Rac1P29S peptide epitope, a necessity not always given for *in silico* predicted neoepitopes but essential for clinical application in an adoptive T cell transfer setting (31, 32). Nevertheless, the three tested cell lines were recognized by the TCR-transduced T cells with varying efficacy. While the Mel20aI cells were lysed by all three TCR-transduced T cells (22894-Rac2, 5934-Rac1 and A12B20-Rac1), the Mel085-A2 and Mel55 cells were not recognized by A12B20. Due to their role in signaling, cell cycle and migration variable recognition might be caused by intrinsic properties of oncogenic Rac GTPase mutants, such as expression too low to be recognized by lower affinity TCRs.

The two TCRs which performed best in the cytotoxicity assays against the Rac1 mutation were subsequently used for *in vivo* studies. Interestingly, when we stained the TCRs with a mutant Rac1-specific pA2 tetramer, the 22894 Rac2-derived TCR showed higher staining intensity than the 5934 Rac1-derived TCR, suggesting that we isolated a TCR with higher affinity after immunization with a heterologous peptide. For HLA-A*02:01 leucine is the dominant anchor amino acid residue at position 2 (33). Thus, the hydrophobic leucine mutation in Rac2P29L at the primary anchor position 2 of the epitope (34) induces stronger binding to the HLA-A*02:01 molecule than the polar serine mutation in Rac1P29S [NetMHC 4.0, IC_50_ 2.3 nM versus 18.2 nM (**Table 1**)]. The phenomenon of enhancing HLA binding and T cell activation by anchor position modification has already been described as altered peptide ligand immunity where modified peptides may act as super-agonists (35, 36). In line with this finding, 22894 TCR-transduced T cells did also induce stronger regression of Rac1P29S expressing tumors in a syngeneic HLA-A2–transgenic mouse model after adoptive T cell therapy. Furthermore, upon antigen encounter, amplification of 22894 TCR-transduced CD8^+^ T cells was significantly higher when compared to that of the CD8^+^ T cells modified with 5934 Rac1-derived TCR, despite comparable T cell transduction rates and comparable recognition of Rac1P29S^+^ tumor cells *in vitro*. Similar observations *in vivo* have been made with a CDK4R24C-derived human TCR that induced more effective rejection and tumor-specific CD8^+^ T cell amplification when tumors expressed the isogenic CDK4R24L mutation (21). The exclusive selection of antigen-negative tumor cells by the heterologous Rac2-specific T cells that led to tumor relapse in our *in vivo* model argues for a strong T cell pressure. Such a selection phenomenon has also been seen in the clinic, when exclusively tumors relapsed under T cell pressure by Kras-specific T cells that had lost the respective restriction element (37). Since there are several recurrent neoantigens that harbor different mutations in the same hotspot region (e. g. Kras, p53), our finding might support the notion of using a heterologous peptide with higher predicted peptide MHC affinity for immunization to isolate TCRs with higher affinity and *in vivo* efficacy.

Our approach thus describes an *in vivo* affinity maturation that is still controlled by thymic selection and may lower the risk of off-target toxicity (38). As the mouse model, we isolated the TCRs from, only expresses human HLA-A2 and is therefore not tolerant for other HLA molecules, we in addition excluded the possibility of alloreactivity by LCL assays and alanine scans. We found that at least the two TCRs with highest clinical potential (22894 and 5934) were not alloreactive against other HLA expressing cell lines. TCR A12B20 showed alloreactivity against two LCLs and was therefore excluded from further use. The recognition pattern of the TCRs was detected in an alanine scan. For the identical recognition pattern of TCRs 5934 and 22894 we found one epitope derived from the GNAZ protein with an overlapping pattern. However, human cell lines naturally expressing this protein were not recognized and, therefore, we minimized the risk of off-target toxicity.

While *in vitro* the Rac1-specific 5934 TCR-transduced T cells performed slightly better in cytotoxicity and IFNγ release, the Rac2-specific 22894 TCR-transduced T cells expanded significantly better *in vivo* and induced more potent regression of the Rac1P29S expressing tumor cells. Discrepancies between *in vitro* and *in vivo* T cell responses have been described, e.g. when analyzing a TCR recognizing the mutant CDK4 isoforms R24L and R24C (21). Therefore, *in vivo* validation of targets and TCRs in suitable mouse models remains unavoidable so far.

In conclusion, we showed that the recurrent neoepitope Rac1P29S_28-36_ is naturally processed and presented and can be successfully targeted with high-affinity TCR-transduced T cells. Rac1P29S-derived TCR 5934 as well as a heterologous Rac2P29L-derived TCR 22894 elicited cytotoxicity against melanoma cell lines naturally expressing the Rac1P29S mutation and induced regression against Rac1P29S expressing tumors *in vivo*. These data suggest that both TCRs show clinical potential of adoptive T cell therapy in HLA-A2 positive melanoma patients expressing the Rac1P29S mutation and open an avenue for using TCRs raised by a heterologous peptide with higher efficacy.

## Data availability statement

The original contributions presented in the study are included in the article/**Supplementary Material**. Further inquiries can be directed to the corresponding author.

## Ethics statement

All animal experiments were performed according to institutional and national guidelines and regulations and were approved by the Landesamt für Gesundheit und Soziales Berlin (H0086/16 and G0112/16).

## Author contributions

GW designed the study. LI, GP, NG, VS, and AP conducted experiments and provided material. LI, GP, TB, and GW analyzed data and wrote the manuscript. TB and GW are joint last authors. All authors contributed to the article and approved the submitted version.
