# Supplementary material for: Targeting the recurrent Rac1P29S neoepitope in melanoma with heterologous high-affinity T cell receptors
Source: Front Immunol. 2023 Feb 16;14:1119498. doi: 10.3389/fimmu.2023.1119498 (PMC9978334; doi:10.3389/fimmu.2023.1119498)
Supplement: Supplementary file 1 [file DataSheet_1.pdf]

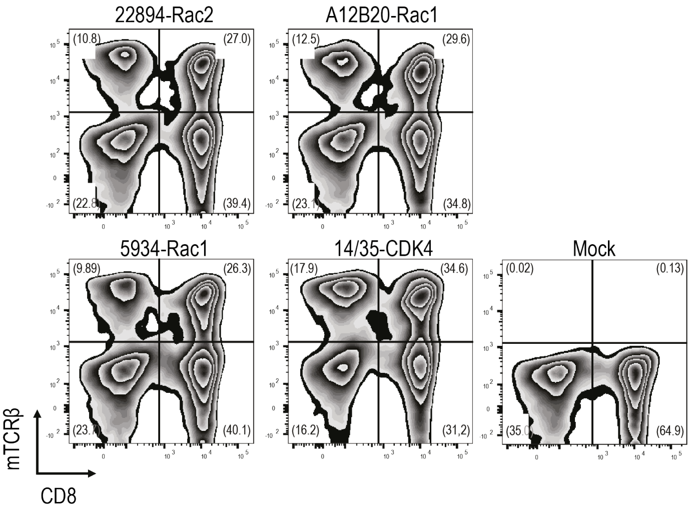

Supplementary Figure 1. TCRs were successfully transduced into human T cells. TCR transduction rates were determined by staining of the mouse TCRβ constant chain and subsequent analysis by flow cytometry. Mock transduced cells are untransduced with an empty virus, 14/35 TCR-transduced T cells serve as a positive control targeting CDK4R24L mutation. Percentages displayed are based on pregated CD3^{+} lymphocytes.

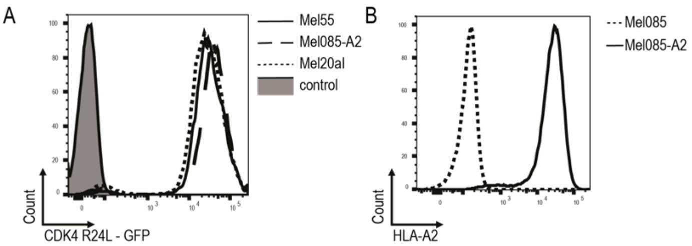

Supplementary Figure 2. Recombinant CDK4R24L and HLA-A*02:01 expression in human melanoma cell lines. (A) GFP intensities of melanoma cell lines after retroviral transduction with CDK4R24L full-length cDNA measured by flow cytometry. Control cells were representative examples of untransduced, GFP negative Mel55 cells. **(B)** The HLA-A*02:01 negative cell line Mel085 was retrovirally transduced with HLA-A*02:01 (Mel085-A2). Expression was confirmed by flow cytometry.

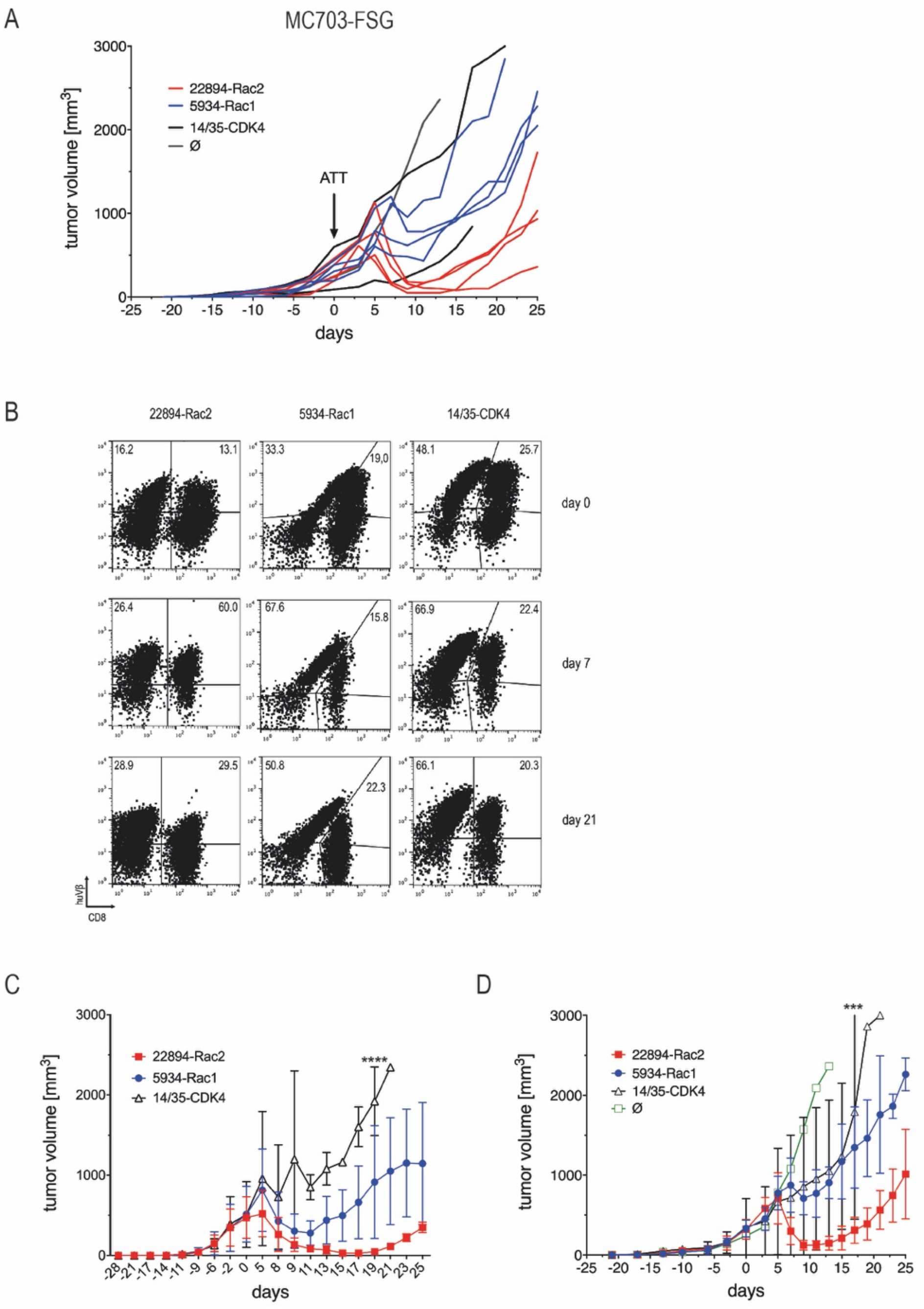

Supplementary Figure 3. Rapid amplification of mutant Rac2- but not Rac1-specific TCR gene-modified T cells upon recognition of Rac1P29S$^+$ tumor cells *in vivo*. (A) 1x10^6 MC703-FSG cells were injected into HHDxRag$^{-/-}$ mice. Mutant Rac1/2-specific T cells were injected (arrow) at an average tumor size of 322 mm^3 (n=4, 22894-Rac2, red lines), 331 mm^3 (n=4, 5934-Rac1, blue lines) and 345 mm^3 (n=3, 14/35-CDK4, black lines), respectively. One mouse did not receive T cells and served as negative control (grey line). Standard deviation (SD) is shown in D. **(B)** Mutant Rac2- but not Rac1-specific TCR gene-modified T cells show rapid amplification upon recognition of Rac1P29S$^+$ FSG-GFP tumor cells. TCR 22894, 5934 and 14/35-transduced T cells were identified by staining with anti-human Vβ22, Vβ9 αnd Vβ1 antibodies, respectively, and number of CD8$^+$/huTCR$^+$ T cells within the adoptively transferred CD3$^+$/huTCR$^+$ T cells was calculated. Numbers give percentage of CD3$^+$/huVβ$^+$ T cells, a representative FACS plot of each treatment group is shown. **(C), (D)** Standard deviation (SD) in growth curves of MC703-FSG cells injected into HHDxRag$^{-/-}$ mice after T cell treatment (ATT) as shown in Figure 3B and Supplementary Figure 3A, respectively. Tumor rejection was compared at the indicated time point using two-way ANOVA.

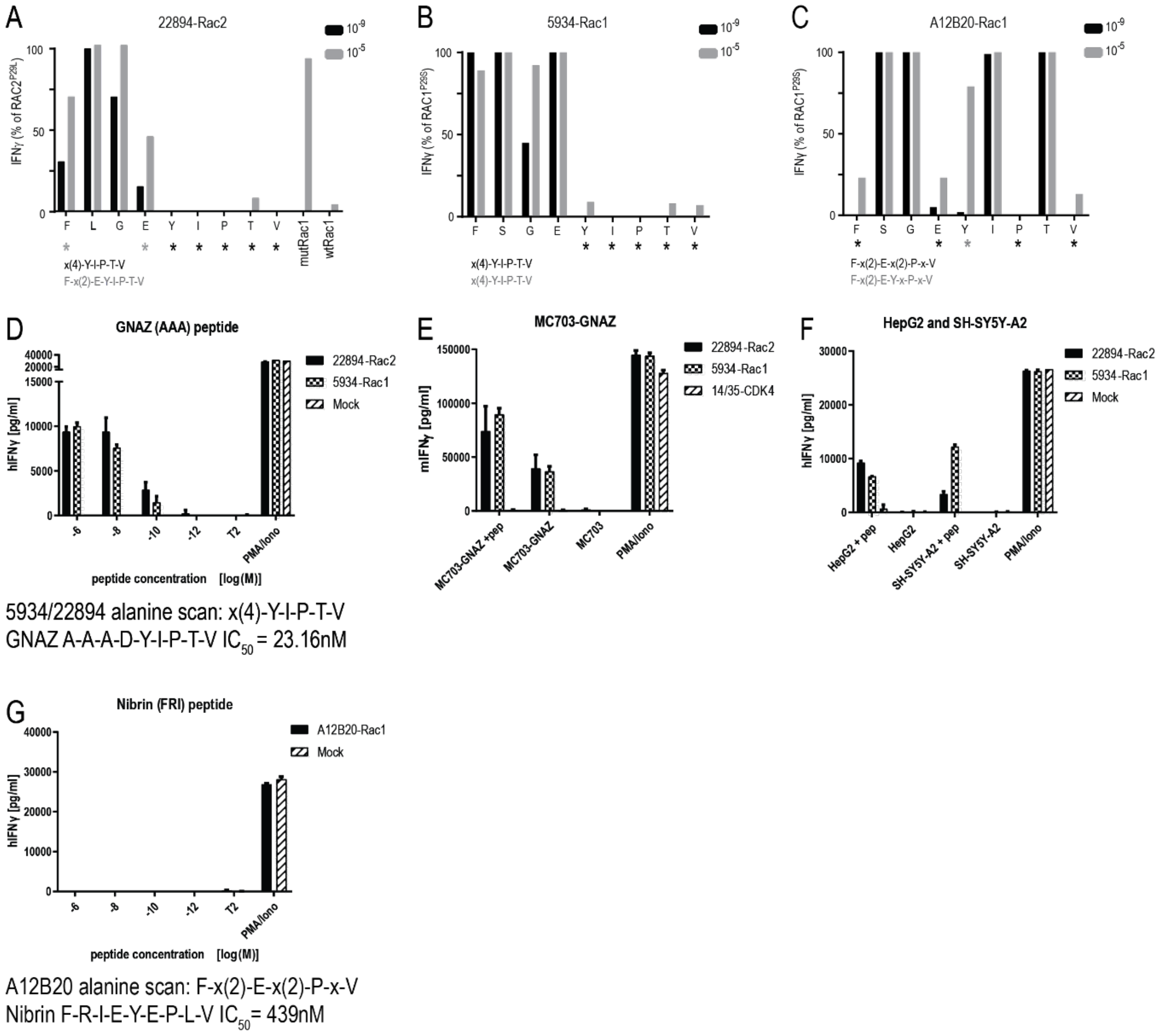

Supplementary Figure 4. Recognition pattern of TCRs determined by alanine scan. (A-C) TCR-transduced human CD8 T cells were co-cultured with T2 cells that were loaded with mutant Rac1/2 peptide containing single alanine exchanges at 10^{-9} M (black bars) and 10^{-5} M (grey bars). After overnight incubation, IFNγ was measured in the supernatant. An amino acid was rated as TCR recognition site when the response to the respective alanine exchanged peptide was less than 50% as compared to the unchanged Rac1/2 peptide. **(D)** TCR-transduced human $CD8^+$ T cells were co-cultured with T2 cells that were loaded with GNAZ (AAA) peptide. **(E)** TCR-transduced mouse $CD8^+$ T cells were co-cultured with MC703 cells cells that were transduced to express a GNAZ triple 35mer. Cells were loaded with 10^{-6} M GNAZ peptide as a positive control. Mutant CDK4 14/35-TCR transduced T cells served as a negative control. **(F)** TCR-transduced human $CD8^+$ T cells were co-cultured with HepG2 and SH-SY5Y-A2 cell lines endogenously expressing GNAZ. Cells were loaded with 10^{-6} M GNAZ peptide as a positive control. **(G)** TCR-transduced human $CD8^+$ T cells were co-cultured with T2 cells that were loaded with Nibrin (FRI) peptide. PMA and Ionomycin (P/I) stimulation served as a positive control. The experiment was performed two times with similar results and graphs represent means of triplicate cultures ±SD.

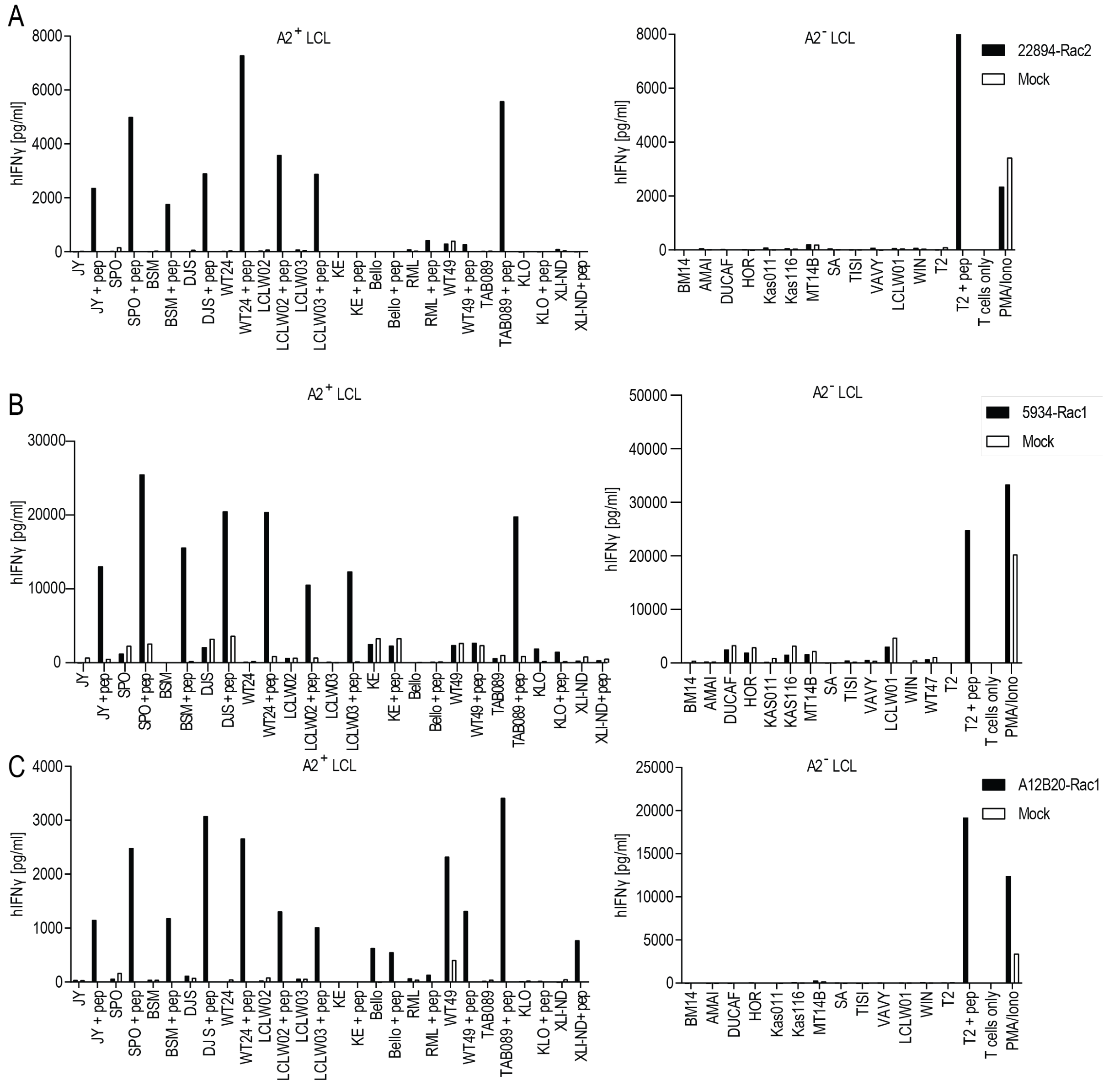

Supplementary Figure 5. No alloreactivity of mutant Rac1/2 TCR-transduced human T cells. (A-C) TCR-transduced $CD8^+$ human T cells (**A**: 22894-Rac2, **B**: 5934-Rac1, **C**: A12B20-Rac1) were co-cultured with a panel of EBV-transformed lymphoblastoid B cell lines (LCLs) expressing different MHC class I molecules (Supplementary Table 1). After overnight incubation IFNγ was measured in the supernatant. A2 positive LCLs are shown with and without peptide at a concentration of 10^{-6} M. PMA and Ionomycin (P/I) stimulation served as positive control. The results are representative of two independent experiments performed with PBLs from different donors.

Supplementary Table 1. List of LCL cell lines.

B-LCL	HLA - A*		HLA - B*		HLA - C*	
SPO	02:01		44:02		05:01	
Bello	02:02	11:01	41:01	52:01	12:02	17:01
LCLW02	02:01	26:01	38:01	44:02	05:01	12:03
LCLW03	02:01	23:01	15:01	58:01	03:04	07:01
TAB089	02:07		46:01		01:02	
WT49	02:05:01		58:01:01		07:18	
XLI-ND	02:10	30:01	13:02	40:06:01:01	06:02	08:01
KLO	02:08	01:01:01:01	08:01:01	50:01:01	07:01:01:01	06:02:01:02
BSM	02:0101		15:010101		03:04:01	
KE	02:01	29:02	44:03	44:05	02:02	16:01
DJS	02:01	03:01	35:01	37:02	04:01	06:02
WT24	02:01:01		27:05:02		02:02:02	
JY	02:01		07:02		07:02	
KAS011	01:0101		37:01		06:02	
BM14	03:01		07:02		07:02	
MT14B	31:01		40:01		03:04	
SA	24:02:01:01		07:02:01		07:02	
HOR	33:0301		44:03:01		14:03	
TISI	24:02:01:01		35:08		04:01	
WIN	01:01		57:01:01		06:02	
KAS116	24:02:01:01		51:01		12:03	
DUCAF	30:02		18:01		05:01	
AMAI	68:02		53:01		04:01	
VAVY	01:01		08:01		07:01	
WT47	32:01		44:02		05:01	
LCLW01	03:01	24:02	15:01	35:01	03:03	04:01
